# Non‐HLA Antibody‐Associated Hyperacute Allograft Rejection Following Lung Transplantation

**DOI:** 10.1111/petr.70457

**Published:** 2026-09-23

**Authors:** Justyna A. Karolak, Ernestina Melicoff, Nidhy P. Varghese, Przemyslaw Szafranski, Esra Yıldız Bölükbaşı, Erin E. McHugh, Sarah Nicholas, Peter Jindra, John Hicks, Eric D. Austin, Paweł Stankiewicz, George B. Mallory

**Affiliations:** ^1^ Chair and Department of Genetics and Pharmaceutical Microbiology Poznan University of Medical Sciences Poznan Poland; ^2^ Department of Molecular and Human Genetics Baylor College of Medicine Houston Texas USA; ^3^ Division of Pediatric Pulmonology, Department of Pediatrics Baylor College of Medicine and Texas Children's Hospital Houston Texas USA; ^4^ Division of Pediatric Pulmonary and Sleep Medicine, Department of Pediatrics University of Minnesota Minneapolis Minnesota USA; ^5^ Section of Allergy and Immunology, Department of Pediatrics Baylor College of Medicine and Texas Children's Hospital Houston Texas USA; ^6^ Department of Surgery, Immune Evaluation Laboratory Baylor College of Medicine Houston Texas USA; ^7^ Department of Pathology Baylor College of Medicine and Texas Children's Hospital Houston Texas USA; ^8^ Division of Pediatric Pulmonary Medicine, Department of Pediatrics Vanderbilt University Nashville Tennessee USA

**Keywords:** developmental lung disease, hyperacute allograft rejection, lung transplantation, pulmonary hypertension, *TBX4* variant

## Abstract

**Background:**

Pediatric‐onset pulmonary hypertension (PH) is a rare, progressive pulmonary vascular disease associated with high mortality. In cases unresponsive to pharmacological therapy with a severe cardiorespiratory phenotype, lung transplantation (LT) remains the final treatment option. Although LT for pediatric PH is considered a procedure with elevated risk, it has been successfully performed in this population.

**Case Presentation:**

A 3‐year‐old boy underwent uncomplicated LT for fulminant *TBX4*‐associated PH and developed hyperacute allograft failure requiring plasmapheresis, augmentation of immunosuppression, and the use of a monoclonal complement inhibitor. Despite extensive treatment the patient died 46 days after LT. Retrospective analysis of the serum sample taken at time of transplant prior to reperfusion was positive for six non‐histocompatibility antibodies: Sjogren syndrome antigen B, thyroglobulin, glutathione S‐transferase Theta‐1, colony stimulating factor 2, intracellular adhesion molecule 1, and interferon gamma that were not present in other pediatric thoracic transplant recipients.

**Conclusions:**

Hyperacute allograft rejection is extremely rare in LT recipients and its occurrence in this patient implies the need for further characterization of non‐HLA antibody‐associated LT rejection.

**Abbreviations**
ASD: atrial septal defect
CMA: chromosomal microarray
CNV: copy‐number variant
CT: computed tomography
HAR: hyperacute allograft rejection
HLA: histocompatibility complex antibodies
LLDD: lethal lung developmental disorder
LPM: liters per minute
LT: lung transplantation
MFI: mean fluorescent intensity
mPAP: mean pulmonary artery pressure
PH: pulmonary hypertension
POD: post‐op day
PRA: panel reactive antibody
PVRi: indexed pulmonary vascular resistance
VV‐ECMO: venous–venous extracorporeal membrane oxygenation
WGS: whole‐genome sequencing
WU: woods units

## 1 Introduction

Pulmonary hypertension (PH) is a rare vasculopathy in infants and children characterized by progressive remodeling of the small pulmonary arteries, resulting in elevated pulmonary pressure and pulmonary vascular resistance [1]. Recent investigations note a significant role for rare genetic variations in a proportion of PH with *TBX4* as the second most common genetic cause of pediatric PH after *BMPR2* [2].

Pediatric‐onset PH may exhibit a limited response to pharmacological therapy leading to progressive right heart failure [3, 4]. In cases with fulminant PH associated with end‐stage lung disease, lung transplantation (LT) is considered as an only treatment option. Reports have described successful LT in a population of pediatric PH patients with post‐operative survival comparable to those observed in pediatric lung transplant recipients with other underlying diseases [5, 6]. Although chronic allograft dysfunction is a common progressive long‐term complication of LT [7], hyperacute allograft rejection (HAR) occurring in the immediate post‐operative period is extremely rare and has been reported only in a few lung transplant patients [8, 9, 10, 11, 12, 13, 14, 15].

Here, we describe a 3‐year‐old boy with PH secondary to a recurrent ~2.2 Mb copy‐number variant (CNV) deletion on chromosome 17q23.1q23.2 involving *TBX4*, who underwent LT complicated by HAR mediated by non‐HLA antibodies.

## 2 Materials and Methods

### 2.1 Ethics Statement

All protocols used in this study were reviewed and approved by the Institutional Review Board for Human Subject Research at Baylor College of Medicine (H‐8712). In compliance with the Declaration of Helsinki, informed written consent for genetic study was obtained for the participated individual.

### 2.2 Case Presentation

The patient was a former full‐term infant who required venous–venous extracorporeal membrane oxygenation (VV‐ECMO) at birth for acute respiratory failure due to persistent PH of the newborn. Because of the severity of illness, lung biopsy was done but was unrevealing of etiology for clinical findings. Initial cardiac catheterization done at 4 months of age showed mild PH (mean pulmonary artery pressure [mPAP] 24 mmHg, indexed pulmonary vascular resistance [PVRi] 2.13 Woods units × m^2^ [WU*m^2^]) and a large patent ductus arteriosus, which was successfully occluded. One month later, the patient had an abrupt cardiopulmonary decline, deemed due to PH crisis, which prompted repeat catheterization with balloon atrial septostomy. He was then transferred to Texas Children's Hospital for PH consultation and therapeutic stabilization. The patient was discharged home at 9 months of age on sildenafil, bosentan, subcutaneous treprostinil, and low flow nasal cannula oxygen supplementation [0.5–0.75 L per minute (LPM) at night to maintain saturation > 90%]. Oxygen needs progressed to ultimately needing bilevel positive airway pressure and high flow nasal cannula although there was little ventilation requirement (pCO_2_s were in the 40–50). Serial echocardiograms demonstrated predominantly left to right shunt via atrial septal defect (ASD) and flattened inter‐ventricular septum with preserved right ventricular function.

Following the initial discharge, the child had frequent admissions for acute‐on‐chronic hypoxemic respiratory failure. Time at home in between hospitalizations was increasingly short and he required escalating levels of oxygen supplementation. He had three bradycardic arrests requiring chest compressions.

**FIGURE 1:**
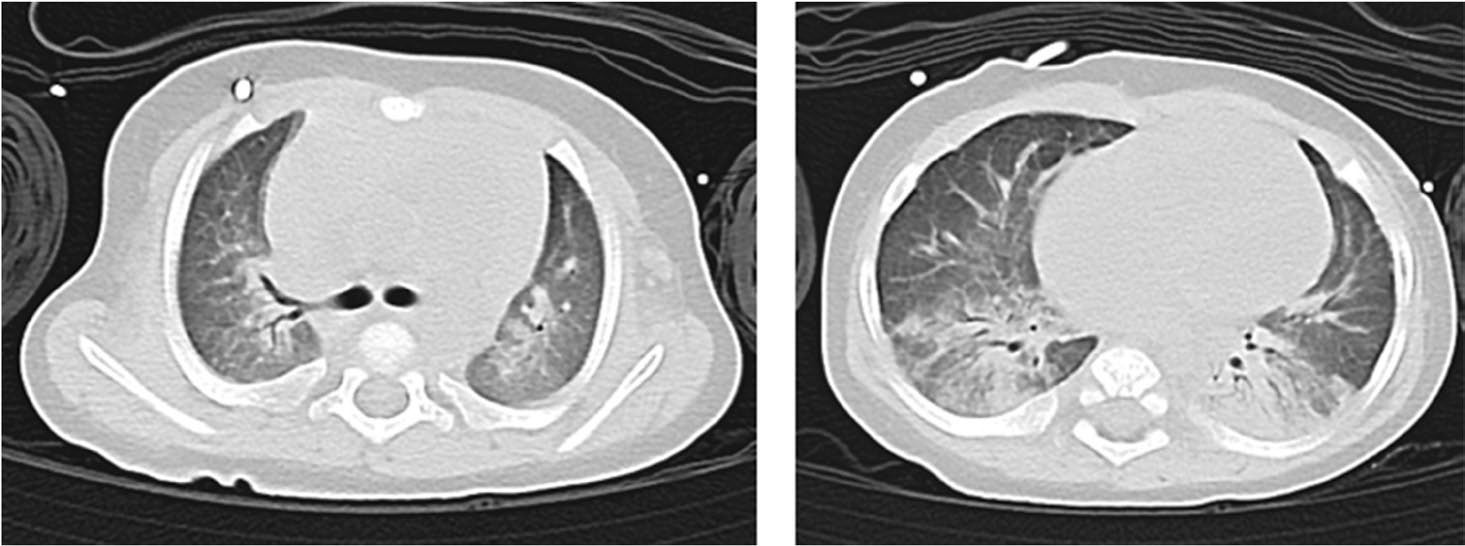
Non‐contrast chest computed tomography (CT) imaging. Diffuse bilateral lower lobe and central ground glass opacities with interlobular thickening and lower lobe consolidation.

At 3 years of age, the child was re‐referred to Texas Children's Hospital for LT evaluation. Chest computed tomography (CT) imaging showed accentuated ground glass densities in dependent portions of both lungs with mild mosaic attenuation (Figure 1). Repeat cardiac catheterization demonstrated progression of disease with elevated mPAP (32 mmHg) and PVRi (6.42 WU*m^2^). The patient was on 6 LPM O_2_, inhaled and oral steroids, diuretics, and supplementation, and after a comprehensive multidisciplinary evaluation, he was approved for listing for LT. Notably, the panel reactive antibody (PRA) screen at the time of listing for LT was negative for Class I and II anti‐HLA antibodies.

**FIGURE 2:**
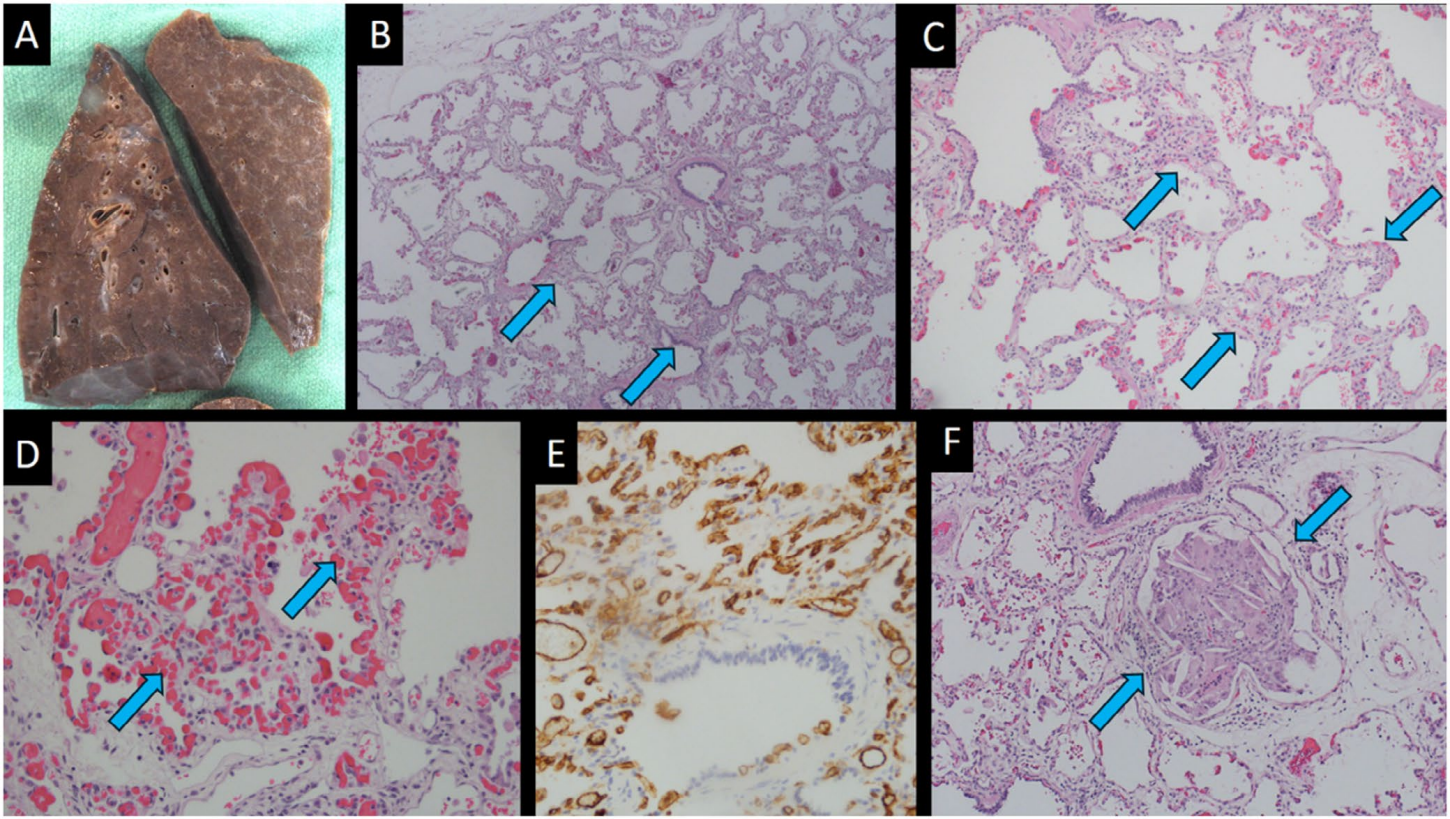
Explanted native lung with features of congenital acinar dysplasia and capillary hemangiomatosis. (A) Cross‐section of right lung with prominent congestion as noted by deep red‐brown character and lung weight of 197 g (expected 88 g for age); (B, C) lung parenchyma with features of acinar dysplasia characterized by simplified lobular and acinar architecture with markedly thickened (arrows) interstitial septa and alveolar walls resulting in poorly formed airspaces (H&E stain); (D, E) exuberant capillary proliferation with intra‐alveolar tufts and capillary walls (arrows), consistent with capillary hemangiomatosis (D: H&E stain; E: CD31 immunohistochemical stain); (F) giant cells intermixed with cholesterol clefts and muciphages (arrows) noted within and expanding the lumen, consistent with lipoid pneumonia (H&E stain).

After 5 weeks on the waiting list, the patient underwent uncomplicated bilateral LT and ASD closure with favorable assessment of the donor organ at implantation and reperfusion. Routine pre‐operative PRA testing and virtual crossmatch were negative. Per protocol, the recipient received intravenous thymoglobulin as induction prior to reperfusion. Cardiopulmonary bypass time was 339 min, and organ ischemic time was 358 min, consistent with institutional norms. The patient received three units of packed red blood cells, two units of fresh frozen plasma, and 10 mL/kg of platelets. The native lung (explant) was notable for severe growth abnormalities with distal, simplified acinar spaces with features corresponding with congenital acinar dysplasia and capillary hemangiomatosis, consistent with the phenotypes observed in patients with *TBX4*‐related abnormalities (Figure 2).

**FIGURE 3:**
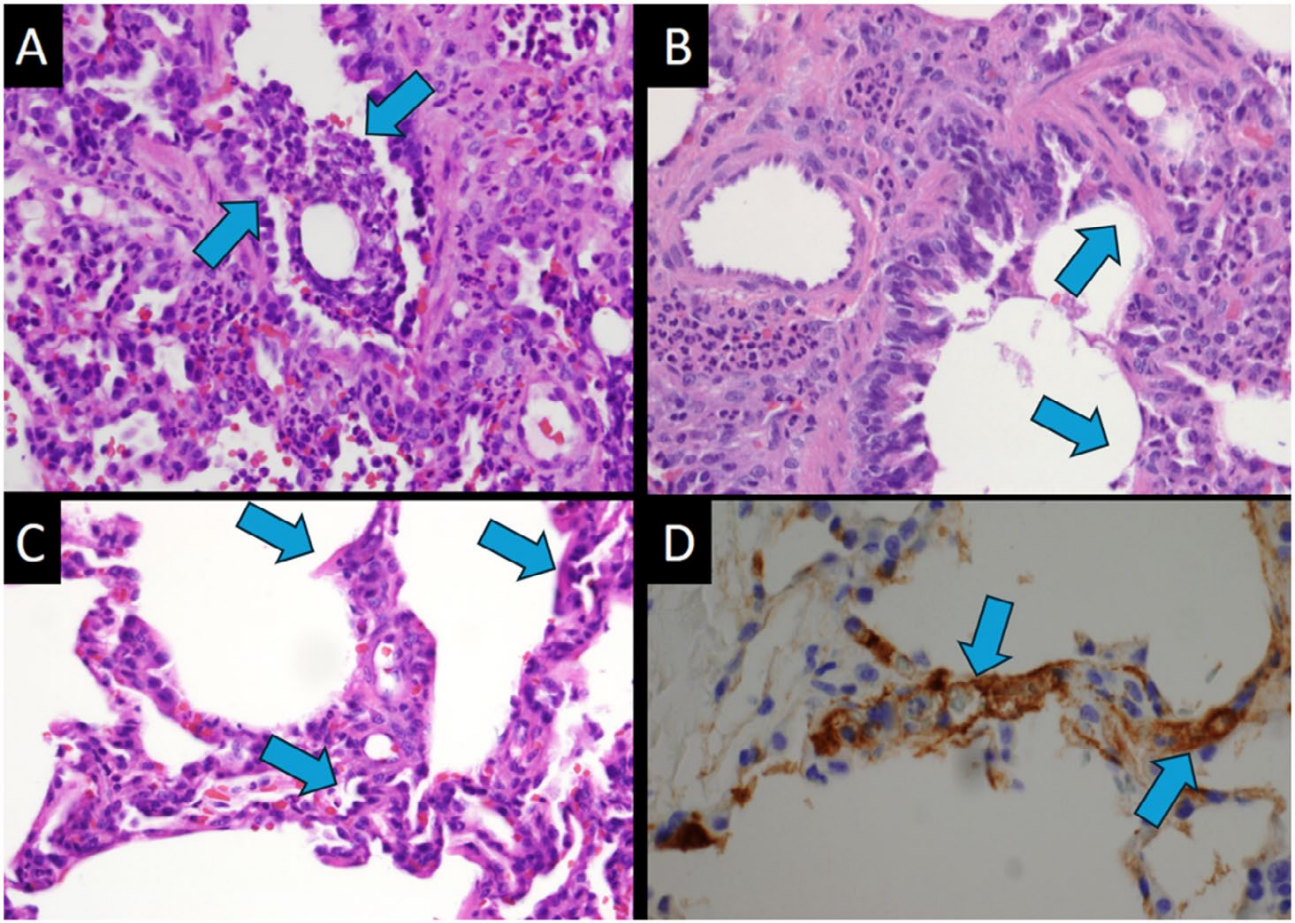
Post‐operative wedge biopsy evaluation. Specimen is consistent with hyperacute rejection. (A, B) Peribronchial and perivascular acute inflammatory infiltrate with acute inflammatory cells (arrows) within bronchiole (A) and partial loss of bronchial epithelium (arrows, B) with acute inflammatory infiltration (H&E stain); (C) extensive interstitial infiltration of alveoli interstitium and capillaritis (arrows, H&E stain); (D) diffuse and strong C4d immunoreactivity (arrows) with alveolar capillaries (C4d immunohistochemical stain).

On arrival in the intensive care unit, initial lung compliance and gas exchange were normal, but the patient's clinical condition worsened rapidly and by 12 h post‐surgery, he required cannulation onto central VV‐ECMO, vasopressor support and modified chronic renal replacement therapy. The tentative diagnosis was Grade 3 primary graft dysfunction. On the 6th post‐op day (POD 6), the patient was taken to the OR for chest closure, transition to peripheral VV‐ECMO cannulation and an elective left lung wedge biopsy. Lung histopathology was consistent with HAR, indicating the high presence of preformed circulating antibodies (Figure 3). Repeat PRA testing on POD7 showed an elevated Class II PRA of 11% with low titer de novo donor specific antibodies against DQ2 at 1261 mean fluorescent intensity (MFI) (Figure S1), suggesting these were produced after lung injury from HAR and not a cause of the complication. Aggressive therapeutic intervention was instituted with plasmapheresis, high dose intravenous immunoglobulin and eculizumab in addition to maintenance immunosuppression with tacrolimus, mycophenolate mofetil and corticosteroids. The patient improved and was supported by high flow oxygen therapy and non‐invasive ventilation.

**FIGURE 4:**
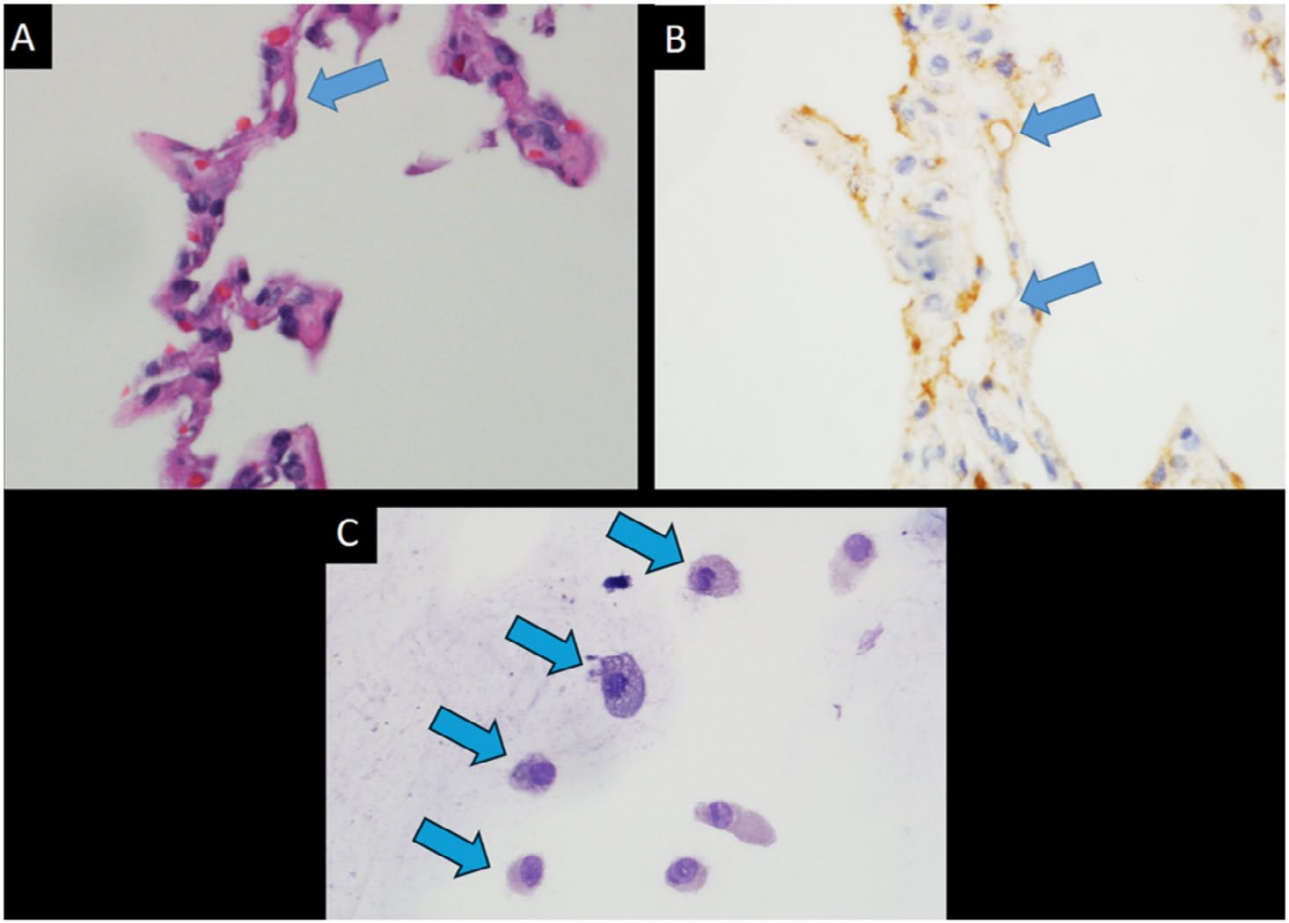
Transbronchial biopsy (A, B) showing a decreased interstitial inflammation and no capillaritis (arrow) and marked diminution in intensity of C4d complement deposition (arrows) with interstitial capillaries (A: H&E stain; B: C4d immunohistochemical stain); (C) Bronchoalveolar lavage comprised of abundant alveolar macrophages (arrows, H&E stain).

Transbronchial biopsy on POD 26 revealed a marked diminution in signs of antibody and complement deposition, and bronchoalveolar lavage showed a macrophage‐predominant specimen (cellular differential: 98% macrophages, 1% neutrophils, and 1% lymphocytes) (Figure 4). Anti‐HLA antibodies tested POD 34 were DSA negative (Figure S2).

**FIGURE 5:**
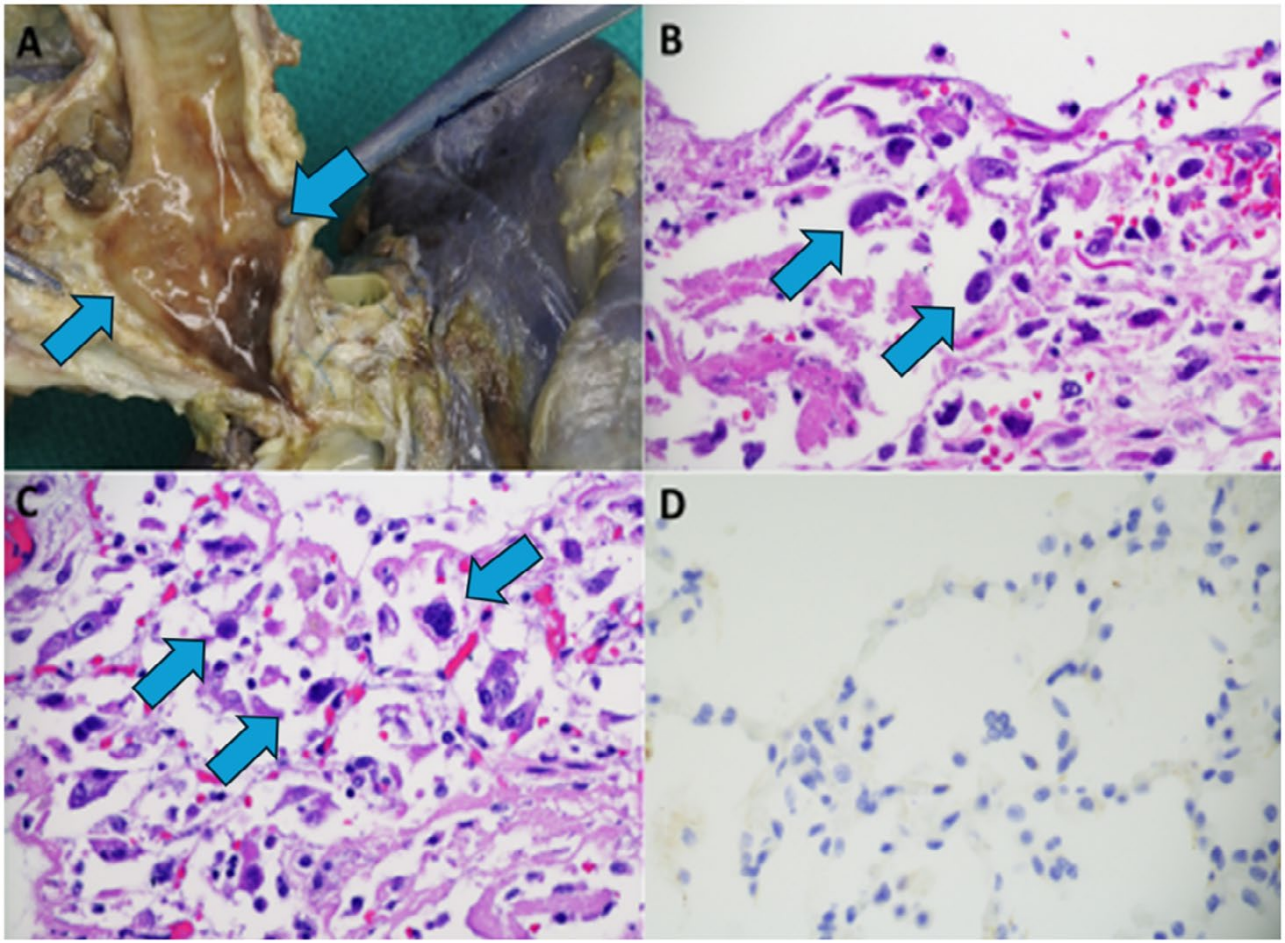
Lung tissue evaluation at autopsy. Anastomotic sites were intact but markedly hyperemic (arrows) at autopsy (A). Photomicrographs of the right mainstem bronchus (B) and lung parenchyma (C) show marked viral cytopathic effect (arrows) consistent with adenovirus infection, confirmed via immunostaining. There was no evidence of antibody mediated rejection at autopsy, including C4d immunostaining negativity (D).

VV‐ECMO decannulation was performed on POD 28. However, the recipient developed fulminant adenoviral sepsis in the following week, and life‐sustaining care was withdrawn 46 days after LT. Transplanted lung autopsy showed acute inflammation and necrotizing bronchitis/bronchiolitis. The overall autopsy findings were consistent with severe Adenovirus pneumonia without detectable evidence of allograft rejection (Figure 5).

### 2.3 Immunological Studies

PRA‐testing in patient's serum and autopsy‐derived tissue was examined before, immediately after transplantation, and post‐mortem. A stored sample of the pre‐transplant serum POD 0 was tested on a commercial testing platform for non‐HLA antibodies (LIFECODES Non‐HLA antibody kit, Immucor, Norcross, GA). Anti‐HLA antibodies were tested by LABScreen Single Antigen Class I and II beads (ThermoFisher One Lambda, West Hills, CA, USA) at POD 7 and 34. For antibodies with low MFI, the anti‐HLA antibodies were tittered (Neat, 1:8, 1:16, 1:32) per standard post‐transplant protocol. Non‐HLA antibodies were considered positive when the background corrected MFI value is above the given manufacturer determined cutoff, and the MFI ratio was equal to or above 1. A clinically significant antibody was defined as one with an MFI ≥ 3000 and titers out between 1:8–1:16, which corresponded to a positive flow crossmatch.

### 2.4 Genetic Studies

DNA and RNA were extracted from the frozen transplanted (AD136) and explanted (AD136N) lung tissues using Gentra Purgene Tissue Kit (Qiagen, Germantown, MD) and miRNeasy Mini Kit (Qiagen), respectively. In addition, RNA was prepared from FFPE age‐matched lung control specimen using Quick‐RNA FFPE Kit (Zymo Res., Irvine, CA). Genomic CNVs in the DNA sample were analyzed at Cook Children's Hospital using chromosomal microarray analysis (CMA). Whole‐genome sequencing (WGS) was performed in DNA samples from both explanted and transplanted lungs using a TruSeq Nano DNA HT Library Prep Kit (Illumina, San Diego, CA) and the HiSeqX platform (Illumina) with 30X coverage as previously described [16].

RNA samples from explanted lungs were reverse‐transcribed using SuperScript III First‐Strand Synthesis System (Invitrogen, Waltham, MA). The transcript levels of *TBX4*, *TBX2*, *FGF10, TMEM100*, and *SLC6A4* involved in lung development, as well as *CXCL1* chemokine (C‐X‐C motif) ligand 1, *CXCL8* (IL8) C‐X‐C motif chemokine ligand 8 (interleukin 8), and *IL7R* from the interleukin signaling pathway, were measured by RT‐qPCR and compared with their counterparts from a control age‐matched normal lung tissue using CFX Connect Real Time System (BioRad, Hercules, CA) and TaqMan assays (Applied Biosystems, Waltham, MA). For relative quantification of the transcript/cDNA, the comparative ∆∆*C*_t_ method with *GAPDH* as an endogenous reference was used. Transcript levels were shown as the fold change in the expression of a selected gene in the patient's lungs relative to the expression of that gene in age‐matched normal lungs.

## 3 Results

### 3.1 Immunological Studies

**FIGURE 6:**
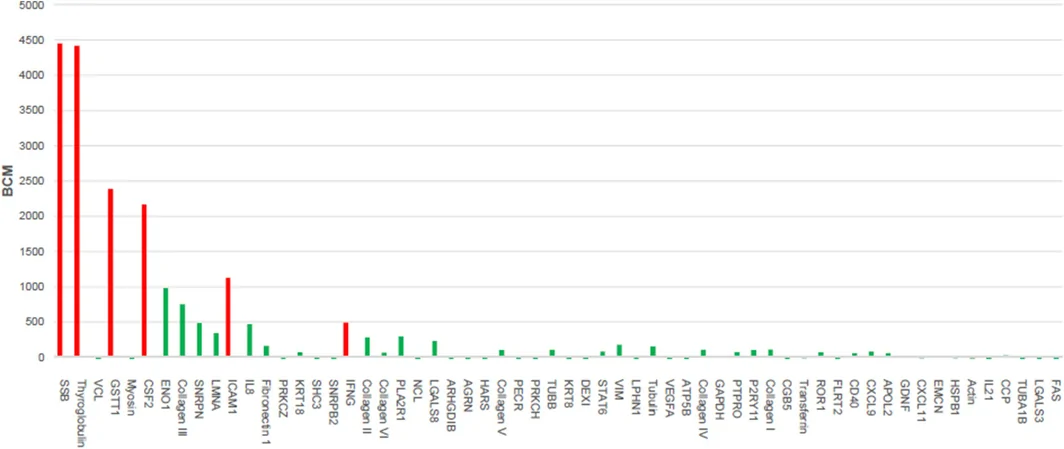
Non‐HLA antibody test pre‐transplant POD 0. Sixty antigen panel to detect non‐HLA antibodies in patient serum. Positive assignments are in red and negative assignments are in green. BCM, background corrected MFI.

The results of the postmortem non‐HLA antibody screening performed in the stored sample of the pre‐transplant serum were positive for six antibodies in descending order: Sjogren syndrome antigen B (SSB), thyroglobulin, glutathione S‐transferase Theta‐1 (GSTT1), colony stimulating factor 2 (CSF2), intracellular adhesion molecule 1 (ICAM‐1), and interferon gamma (IFNG) (Figure 6, Table S1). Of all six elevated antibodies, SBB and thyroglobulin are considered as clinically significant (MFI ≥ 3000). None of these antibodies were elevated beyond a standard background‐corrected negative control in four other pediatric thoracic transplant patients with 0% PRA (Table S2).

### 3.2 Genetics Analyses

CMA and WGS of the explanted lungs revealed a recurrent ~2.2 Mb CNV deletion, involving *TBX4* and its putative lung‐specific super‐enhancer region on chromosome 17q23.1q23.2 (Figure S3).

WGS analysis of the DNA sample from the transplanted lung revealed approximately 25%–30% somatic mosaicism for this CNV deletion (Figure S3).

RNA analyses showed that *TBX4*, *TBX2*, *FGF10, TMEM100*, *SLC6A4*, *CXCL8* (*IL8*), *IL7R*, and *CXCL1* transcripts were downregulated in the native lung when compared to normal control (Figure S4).

## 4 Discussion

Hyperacute allograft rejection is not infrequently fatal and long‐term outcomes after all forms of antibody‐mediated allograft rejection are suboptimal [17]. HAR was initially described in renal and heart transplantation in the 1960s [18, 19]. However, the first reported HAR case in a LT recipient dates back to 1996 [8], with eight additional cases described in subsequent years [9, 10, 11, 12, 13, 14, 15]. The majority of HAR cases after LT derive from circulating anti‐donor specific HLA that lead to rapid and severe injury to the allograft. More recently, however, instances of non‐HLA antibodies causing HAR have been reported [15], prompting pre‐transplant screening of recipients for PRA as a routine practice. This far, all documented LT recipients who developed HAR were adults, except for our patient [8, 9, 10, 11, 12, 13, 14, 15] with only three cases being successfully treated [11, 14].

PRA‐testing in the patient's serum was negative before and immediately after transplantation. However, a wedge biopsy of the transplanted lung revealed severe antibody‐mediated HAR with heavy complement deposition in the lung tissue. The development of HAR was rapid and severe, prompting our team to do further evaluation of underlying genetics and immunochemistry. Retrospective analysis of the serum taken at time of transplantation prior to reperfusion showed the presence of non‐HLA antibodies against SSB, thyroglobulin, GSTT1, CSF2, ICAM‐1, and IFNG. These antibodies were not detected in four other pediatric thoracic transplant recipients aged 1–11, suggesting that this finding is not incidental, but rather clinically meaningful. Although the role of elevated levels of these six antibodies in the development of HAR in the presented patient remains unclear, some of them have been previously associated with acute rejection or chronic allograft dysfunction after thoracic transplantation.

It has been shown that higher level of anti‐thyroglobulin autoantibodies is associated with an elevated risk of chronic lung allograft dysfunction [20]. In contrast, the presence of pre‐existing antibodies against GSTT1 before both heart or lung transplantation is associated with an increased risk of acute allograft rejection [21]. Of note, the presence of pre‐transplant anti‐GSST1 antibodies in patients with GSTT1‐null genotype could be explained by platelet transfusion performed before the surgery, as described before [21]. In cardiac transplantation, SSB has emerged as a novel non‐HLA rejection target that synergizes with donor‐specific HLA antibodies leading to a higher risk of allograft rejection compared with donor‐specific HLA antibodies alone [22]. Leukocyte adhesion molecules like ICAM‐1 and its ligands are known mediators of lung inflammation [23] and blockade of ICAM‐1 early after allogeneic LT in mice model, prevents acute cellular rejection [24]. Antibodies against CSF2 have been previously found to be associated with development of HAR following kidney transplantation [25], but not after LT.

Among non‐HLA antibodies implicated in thoracic transplantation but absent in our patient, anti‐enolase, anti‐collagen, anti‐angiotensin II type 1 receptor (AT1R) or anti‐vimentin antibodies in LT [26] as well as antibodies against AT1R or MHC Class I‐related chain A (MICA) in heart transplant recipients [21, 27], have been associated with antibody‐mediated rejection and chronic allograft injury rather than HAR.

Interestingly, there is some evidence in the literature pointing to the potential influence of genetic factors on allograft rejection. In addition to the above mentioned mismatch in GSTT1 locus [21], recent studies have revealed that the recipients with homozygous 1.5 kb CNV deletion downstream of *LIMS1* locus were at a higher risk of acute kidney transplant rejection, due to the presence of anti‐LIMS1 antibodies, when receiving a transplant from a donor with at least one normal allele [28]. A genomic collision within the *LIMS1* locus has been subsequently found in patients with heart transplantations [29]. Although *LIMS1* is also expressed in human lungs, there is no evidence suggesting that *LIMS1* could be a minor histocompatibility antigen associated with lung graft rejection.

In the transplanted lungs on POD 6, we identified 25%–30% mosaicism for the CNV *TBX4* deletion, indicating that a substantial fraction of recipient cells infiltrated the donor lungs and corroborating the possible immune over‐reaction and HAR. Whereas haploinsufficiency of *TBX4* led to the primary patient's lung disease, it is unlikely that it has contributed to HAR. Among other genes located in the deleted region is *PPM1D* (also known as *WIP1*), a serine/threonine protein phosphatase. *Wip1* deficiency in mice was shown to lead to the enhanced activity and migration ability of neutrophils [30]. In addition, mice with knock‐out of *Wip1* presented with intestinal ischemia/reperfusion injury with increased infiltration of neutrophils, leading to a higher production of cytokines [31]. Although PPM1D is an important mediator of inflammatory response, it is unknown how its deficiency could promote the possible immune over‐reaction observed in our patient.

Interestingly, deletion of 17q23.1q23.2, involving *TBX4*, entails deregulation of several other genes related to the respiratory system, including *TBX2*, *FGF10*, *TMEM100*, and *SLC6A4* or those involved in inflammatory response, *CXCL1*, *CXCL8*, and *IL7R* [32]. Notably, we found these genes downregulated in the native lung of the described patient. Whether their presence in the allograft is causatively associated with incompatibility and non‐HLA‐directed immune activation would need further investigation.

## 5 Conclusions

The implications of our findings suggest that if similar patients become candidates for LT in the future, a thorough genetic and immunologic evaluation should take place pre‐operatively and careful assessment of the patient/allograft must be done in the immediate post‐operative period. Pre‐transplant assessment of non‐HLA antibodies may provide insights into the patient's immune reactivity, and the need for an antibody removal induction protocol even if the strongest antibody specificity recruiting complement is unknown. This patient's HAR was successfully treated, supporting that LT remains a viable option to consider for patients with end‐stage cardiorespiratory disease.

## Funding

This research was supported by NIH/NHLBI grant 1R01HL165301‐01A1 (P. Stankiewicz).

## Conflicts of Interest

Justyna A. Karolak, Ernestina Melicoff, Nidhy P. Varghese, Esra Yıldız Bölükbaşı, Erin E. McHugh, Sarah Nicholas, Peter Jindra, John Hicks, and George B. Mallory have no conflicts of interest. Przemyslaw Szafranski is the investigator on NIH/NHLBI sponsored project. Eric D. Austin serves on Merck's Inc. and Liquidia Inc. Scientific Advisory Boards without direct compensation; he is the Scientific Chair Lead for the not‐for‐profit organization TBX4Life and a Board member for the ClinicalTrials.gov ID NCT04240821 without direct compensation. Paweł Stankiewicz is the principal investigator on NIH/NHLBI sponsored project.

## Supporting information


**Figure S1:** HLA Class II single antigen bead panel. POD 7 highlighting DSA to DQA1*05:01 DQB1*02:01 in the blue box.
**Figure S2:** HLA Class II single antigen bead panel. POD 34 highlighting a previous DSA in the blue box is now negative. No detected anti‐HLA Class II antibodies were DSA.
**Figure S3:** Integrative genomic viewer (IGV) showing the heterozygous recurrent ~2.2 Mb CNV deletion on chromosome 17q23.1q23.2 in the explanted and transplanted lungs. Note ~25%–30% somatic mosaicism in the transplanted lungs, indicating a high level of infiltration of nucleated leukocytes.
**Figure S4:** Relative fold change in expression of the *TBX4*, *TBX2*, *FGF10, TMEM100*, *SLC6A4*, *CXCL8* (*IL8*), *IL7R*, and *CXCL1* genes in the patient explanted lung (AD136N) compared to normal lung control. Data represents means of duplicate experiments (*t* test **p ≤ 0.0001; *p < 0.01).
**Table S1:** The mean fluorescent intensity for the non‐HLA antibodies at the time of lung transplantation.
**Table S2:** The list of non‐HLA antibodies detected in other pediatric thoracic transplant recipients.

## Data Availability

The data that support the findings of this study are available from the corresponding author upon reasonable request.
