# Supplementary material for: Non‐HLA Antibody‐Associated Hyperacute Allograft Rejection Following Lung Transplantation
Source: Pediatr Transplant. 2026 Sep 23;30(9):e70457. doi: 10.1111/petr.70457 (PMC13599712; doi:10.1111/petr.70457)
Supplement: Supplementary file 1 [file PETR-30-e70457-s001.docx]

**SUPPLEMENTAL MATERIAL**

**Non-HLA antibody-associated** **hyperacute allograft rejection following lung transplantation**

**Running Title:** Non-HLA hyperacute lung allograft rejection

Justyna A. Karolak^1,2^, Ernestina Melicoff^3*^, Nidhy P. Varghese^3^, Przemyslaw Szafranski^2^, Esra Yıldız Bölükbaşı^2^, Erin E. McHugh^3,4^, Sarah Nicholas^5^, Peter Jindra^6^, John Hicks^7^, Eric D. Austin^8^, Paweł Stankiewicz^2^, George B. Mallory^3^

*^1^Poznan University of Medical Sciences, Chair and Department of Genetics and Pharmaceutical Microbiology, Poznan, Poland*

*^2^Baylor College of Medicine, Department of Molecular and Human Genetics, Houston, TX, USA*

*^3^Baylor College of Medicine and Texas Children’s Hospital, Division of Pediatric Pulmonology, Department of Pediatrics, Houston, TX, USA*

*^4^University of Minnesota, Division of Pediatric Pulmonary and Sleep Medicine, Department of Pediatrics, Minneapolis, MN, USA*

*^5^Baylor College of Medicine and Texas Children’s Hospital, Section of Allergy and Immunology, Department of Pediatrics, Houston, TX, USA*

*^6^Baylor College of Medicine, Department of Surgery, Immune Evaluation Laboratory, Houston, TX, USA*

*^7^Baylor College of Medicine and Texas Children’s Hospital, Department of Pathology, Houston, TX, USA*

*^8^Vanderbilt University, Department of Pediatrics, Division of Pediatric Pulmonary Medicine, Nashville, TN, USA*

***Corresponding author:** Ernestina Melicoff, Division of Pediatric Pulmonology, Department of Pediatrics, Baylor College of Medicine and Texas Children’s Hospital, Houston, TX, USA

Email address: [exmelico@texaschildrens.org](mailto:exmelico@texaschildrens.org)

**Figure S1. HLA Class II single antigen bead panel.** POD 7 highlighting DSA to DQA1*05:01 DQB1*02:01 in the blue box.

**Figure S2. HLA Class II single antigen bead panel.** POD 34 highlighting a previous DSA in the blue box is now negative. No detected anti-HLA class II antibodies were DSA.

**Figure S3.** **Integrative Genomic Viewer (IGV) showing the heterozygous recurrent ~2.2 Mb CNV deletion on chromosome 17q23.1q23.2 in the explanted and transplanted lungs.** Note ~25-30% somatic mosaicism in the transplanted lungs, indicating a high level of infiltration of nucleated leukocytes.

**Figure S4.** **Relative fold change in expression of the *TBX4*, *TBX2*, *FGF10,* *TMEM100*, *SLC6A4*, *CXCL8* (*IL8*), *IL7R*, and *CXCL1* genes in the patient explanted lung (AD136N) compared to normal lung control**. Data represents means of duplicate experiments (*t* test ***P* ≤ 0.0001; **P* < 0.01).

**Table S1.** The mean fluorescent intensity for the non-HLA antibodies at the time of lung transplantation.

| Antigen | MFI | BCM | Cutoff | Assign |
| --- | --- | --- | --- | --- |
| **SSB** | **5904** | **4450** | **2909** | **POS** |
| **Thyroglobulin** | **5090** | **4419** | **1341** | **POS** |
| VCL | 2961 | -1090 | 8103 | NEG |
| **GSTT1** | **2682** | **2388** | **588** | **POS** |
| Myosin | 2387 | -342 | 5457 | NEG |
| **CSF2** | **2323** | **2166** | **313** | **POS** |
| ENO1 | 2059 | 975 | 2168 | NEG |
| Collagen III | 1677 | 749 | 1856 | NEG |
| SNRPN | 1575 | 484 | 2183 | NEG |
| LMNA | 1339 | 342 | 1994 | NEG |
| **ICAM1** | **1246** | **1129** | **235** | **POS** |
| IL8 | 945 | 469 | 952 | NEG |
| Fibronectin | 945 | 160 | 1569 | NEG |
| PRKCZ | 892 | -72 | 1928 | NEG |
| KRT18 | 743 | 69 | 1348 | NEG |
| SHC3 | 713 | -51 | 1526 | NEG |
| SNRPB2 | 672 | -398 | 2140 | NEG |
| **IFNG** | **655** | **489** | **332** | **POS** |
| Collagen II | 651 | 281 | 740 | NEG |
| Collagen VI | 646 | 64 | 1164 | NEG |
| PLA2R1 | 644 | 295 | 698 | NEG |
| NCL | 640 | -171 | 1622 | NEG |
| LGALS8 | 614 | 224 | 779 | NEG |
| ARHGDIB | 603 | -79 | 1364 | NEG |
| AGRN | 526 | -59 | 1168 | NEG |
| HARS | 500 | -163 | 1326 | NEG |
| Collagen V | 463 | 101 | 724 | NEG |
| PECR | 461 | -161 | 1244 | NEG |
| PRKCH | 451 | -339 | 1580 | NEG |
| TUBB | 450 | 103 | 695 | NEG |
| KRT8 | 443 | -48 | 982 | NEG |
| DEXI | 401 | -63 | 929 | NEG |
| STAT6 | 396.5 | 80.2313 | 632.54 | NEG |
| VIM | 376.5 | 173.256 | 406.49 | NEG |
| LPHN1 | 373 | -201.638 | 1149.3 | NEG |
| Tubulin | 359 | 152.406 | 413.19 | NEG |
| VEGFA | 346 | -59.15 | 810.3 | NEG |
| ATP5B | 342 | -120.663 | 925.33 | NEG |
| Collagen IV | 342 | 103.075 | 477.85 | NEG |
| GAPDH | 341 | 10.1563 | 661.69 | NEG |
| PTPRO | 330 | 72.5625 | 514.88 | NEG |
| P2RY11 | 321.5 | 99.9438 | 443.11 | NEG |
| Collagen I | 314 | 109.656 | 408.69 | NEG |
| CGB5 | 299 | -98.0688 | 794.14 | NEG |
| Transferrin | 282 | -14.0875 | 592.18 | NEG |
| ROR1 | 274 | 71.4438 | 405.11 | NEG |
| FLRT2 | 269.5 | -67.1063 | 673.21 | NEG |
| CD40 | 267 | 58.4125 | 417.18 | NEG |
| CXCL9 | 267 | 82.7688 | 368.46 | NEG |
| APOL2 | 266.5 | 56.7125 | 419.58 | NEG |
| GDNF | 258 | 17.6813 | 480.64 | NEG |
| CXCL11 | 214 | -13.4625 | 454.93 | NEG |
| EMCN | 171 | -6.7 | 355.4 | NEG |
| HSPB1 | 168 | -13.1438 | 362.29 | NEG |
| Actin | 118.5 | -20.2875 | 277.58 | NEG |
| IL21 | 117 | -45.3313 | 324.66 | NEG |
| CCP | 114 | 30.25 | 167.5 | NEG |
| TUBA1B | 106 | -94.775 | 401.55 | NEG |
| LGALS3 | 87 | -156.95 | 487.9 | NEG |
| FAS | 85 | -22.05 | 214.1 | NEG |

Abbreviations: MFI, mean fluorescence intensity; BCM, background corrected MFI. BCM and MFI ratio with the cutoff value defined by the manufacturer (MFI/cutoff value) was calculated for each target antigen. Non-HLA antibodies were considered positive when the background corrected MFI value is above the given manufacturer determined cutoff, and the MFI ratio was equal to or above 1.  Antibodies marked in bold were positive. A clinically significant antibody was considered as one with an MFI ≥3000 and titers out between 1:8 – 1:16, which corresponded to a positive flow crossmatch.

**Table S2.** The list of non-HLA antibodies detected in other pediatric thoracic transplant recipients.

| Sample # | Age | Sex | HLA-Ab/PRA | Non-HLA antibodies |
| --- | --- | --- | --- | --- |
| Patient 1 | 1 y/o | F | 0/0 | None |
| Patient 2 | 11 y/o | F | 0/0 | None |
| Patient 3 | 1 y/o | M | 0/0 | + for Tubulin |
| Patient 4 | 1 y/o | F | 0/0 | + for GAPDH |

Abbreviations: F, female; M, male; y/o, years old
